# Tree Endotherapy: A Comprehensive Review of the Benefits and Drawbacks of Trunk Injection Treatments in Tree Care and Protection

**DOI:** 10.3390/plants14193108

**Published:** 2025-10-09

**Authors:** Alessandra Benigno, Chiara Aglietti, Viola Papini, Mario Riolo, Santa Olga Cacciola, Salvatore Moricca

**Affiliations:** 1 Department of Agricultural, Food, Environmental and Forestry Science and Technology (DAGRI), Plant Pathology and Entomology Section, University of Florence, 50144 Florence, Italy; alessandra.benigno@unifi.it (A.B.); viola.papini@unifi.it (V.P.); salvatore.moricca@unifi.it (S.M.); 2 Department of Agriculture, Food and Environment, University of Catania, 95123 Catania, Italy; mario.riolo@unict.it (M.R.); olgacacciola@unict.it (S.O.C.)

**Keywords:** trunk injection, xylem transport, systemic delivery, pressurized injection, non-pressurized injection, sustainable pest and disease management, eco-friendly farming practices

## Abstract

Tree endotherapy has risen to prominence in the field of precision agriculture as an innovative and sustainable method of tree care, being respectful of both environmental protection and consumer health needs. A comprehensive review of the state of the art of research in this field has made it possible to spotlight the main advantages of tree infusion, which has undergone significant progress in step with technological innovation and an increased understanding of tree anatomy and physiology. The major criticalities associated with this technique, as well as the biological and technical–operational obstacles that still hinder its wider use, are also highlighted. What emerges is an innovative and rapidly expanding technique in tree care, in both the cultivation and phytosanitary management of fruit and ornamental trees. Some of the strengths of the endotherapy technique, such as the next-to-no water consumption, the strong reduction in the use of fertilizers and pesticides, the possibility of using biological control agents (BCAs) or other products of natural origin, the precision administration of the product inside the xylem of the tree, and the efficacy (20–90%) and persistence (1–2 years) of treatments, make it one of the cornerstones of sustainable tree protection at present. With a very low consumption of the “active ingredient”, endotherapy has a negligible impact on the external environment, minimizing the drift and dispersal of the active ingredient and thus limiting the exposure of non-target organisms such as beneficial insects, birds, and wildlife. The large-scale application of the technique would therefore also help to achieve an important goal in “climate-smart agriculture”, the saving of water resources, significantly contributing to climate change mitigation, especially in those areas of the planet where water is a precious resource.

## 1. Introduction

Tree endotherapy, also known as trunk injection, is a technique used for injecting substances directly into tree trunks [1,2]. This method allows for the systemic delivery of compounds such as insecticides [3], fungicides [4], and antibiotics [5] directly into the plant’s vascular system by exploiting xylem transport [6]. Most studies involving endotherapy concerned the application of chemicals for the control of pests and pathogens; however, several recent studies have shown the possibility of applying other product classes (e.g., phytohormones and bioformulations) in order to enhance plant growth and abiotic stress tolerance and induce defense response resistance against pathogenic infections [7] (see Section 5.1). Chemicals have also been used to try to kill trees in order to limit the epidemic spread of diseases. Panconesi [8] attempted to inject sodium hypochlorite and the herbicide glyphosate (alone or in combination) under pressure into the trunks of plane trees growing in rows infected with the pathogen *Ceratocystis fimbriata* f. sp. *platani*, the causal agent of “blue stain canker”, to prevent its spread through root anastomoses between neighboring trees. Solutions like this are nothing new: as early as the 15th century, Leonardo da Vinci [9] attempted to poison apple trees by injecting arsenic into their trunks using a rudimentary drill.

Modern endotherapy, also taking advantage of the increased knowledge of plant physiology principles underlying the absorption and translocation of the active ingredient in the tree, has evolved with advancements in injection devices, shifting from simple hand drills to pressurized systems and lenticular blades designed to minimize tissue damage [10] (see Section 4). At present, endotherapy is considered an environmentally friendly technique within the new farming management concept of “precision agriculture”, which aims to increase efficiency, productivity, and sustainability in agriculture, thus also taking care to maintain ecosystem health and mitigate climate change impacts (see Section 3). In plant protection, the technique has been predominantly employed in the treatment of pathogens and pests affecting perennial crops, such as fruit trees, the type of crop on which the greatest number of studies have been conducted [11]. However, many studies have investigated the possibility of applying endotherapy to protect deciduous and ornamental trees from damaging agents, including invasive organisms [11,12] (see Section 5.3). The increasing adoption of endotherapy, particularly in the management of urban and rural trees, underscores its importance as a promising innovation, offering a potentially vital and ever-evolving approach to tree care, aided also by recent technological advances (see Section 5.2). However, significant challenges remain, including the risk of tissue damage at the injection site, the need for specific protocols for vulnerable tree species, and knowledge gaps regarding aspects like optimal dosages and long-term ecotoxicological effects (see Section 6). The purpose of this review is to provide an overview of endotherapy techniques and how they have evolved as a result of scientific advances, such as those relating to increased knowledge of the structure of the xylem network underlying sap flow dynamics. It will also highlight recent innovations in the precise physical administration of active ingredients and in formulation technologies. Improvements in endotherapy technology will require continued investment in research, the standardization of methodologies, and strong collaboration between scientists, industry, and tree care professionals (see Section 7).

## 2. Advantages of Endotherapy Treatments

Endotherapy is a promising solution to overcome the limitations of traditional pesticide application methods, offering a targeted, efficient, and long-lasting way to protect plants from pathogens and pests [13]. Since the method exploits the natural xylem transport of the plant, it is possible to apply a targeted fight against pathogens and pests, reaching parts of the plant that can be more than 10 m high without the need for specialized equipment [1,6]. Hence, endotherapy can be used to treat large trees or portions of them that are difficult to reach with conventional methods like spraying, and can be applied in difficult operating conditions, where foliar treatments are complex or ineffective [14]. As the products are gradually released into the plant’s vascular system and are protected from external influences (e.g., solar radiation), endotherapy can provide longer-lasting protection [5], translocating active ingredients within the plant tissues and reducing the needed treatment frequencies [15]. This direct delivery ensures that the active ingredient reaches the target quickly and efficiently, bypassing barriers such as the cuticle or roots, and allows for localized treatments that can be applied when lethal or persistence attacks by pests or pathogens are present [16]. Furthermore, the direct injection into the trunk reduces losses due to drift, runoff, leaf wash-off, or degradation by UV radiation [17], ensuring that a higher dose of the product reaches the intended target compared to foliar sprays or soil drenches [6].

## 3. Eco-Friendly and Safety Features

Endotherapy presents a compelling alternative to traditional pesticide application methods by offering significant advantages in reducing environmental pollution, minimizing the risk to non-target organisms, and enhancing the safety of both applicators and the public [18]. For these reasons, it is gaining recognition as a promising alternative to conventional pesticide application methods like spraying and soil drenching in managing pests and diseases in various tree species, including those in orchards, forests, and urban areas [13]. As endotherapy involves the direct introduction of plant protection products into the tree’s vascular system [14], it offers significant advantages in terms of reducing environmental impact and enhancing safety for humans, animals, and the environment in general. Conventional pesticide application methods, particularly spraying, are often associated with substantial off-target losses due to drift [1], with estimations suggesting that a vast majority of applied pesticides (around 99.9% in some cases) may not reach the target pests and instead disperse into the environment [2]. Endotherapy directly addresses this issue by significantly reducing the dispersion of plant protection products into the environment [15,19]. By delivering the active ingredient directly into the sap circulation system, it minimizes or even zeroes out the drift and dispersal of chemicals, thus limiting the exposure of non-target organisms such as beneficial insects, birds, and wildlife [20]. This targeted approach contributes to the ecological resilience of ecosystems by avoiding broad environmental contamination [21]. Furthermore, endotherapy enables the use of lower doses of plant protection products compared to spraying or soil drenching to achieve equivalent or even higher efficacy [2,12]. Because the entire dose is delivered directly into the sap flux, a greater quantity of the product reaches the target site within the tree [11]. This reduced reliance on large quantities of pesticides further lessens the overall environmental burden associated with plant protection [6]. Another critical environmental benefit of endotherapy is that it avoids the contamination of surface and groundwater [22]. Unlike soil drenching, which can lead to leachate and accumulated residues that pollute groundwater [10], or spraying, where runoff can contaminate surface waters, the closed system of trunk injection prevents the release of pesticides into these sensitive water bodies [15]. This is particularly important in urban areas where chemical spraying might be prohibited or restricted due to the proximity to water sources and human populations [14]. Endotherapy also offers enhanced safety for both the operators applying the treatments and the general public. When spraying methods are employed, workers are at a higher risk of exposure to high concentrations of agrochemicals during mixing and application [13]. Endotherapy significantly reduces the exposure of operators to plant protection products as it eliminates the need for the widespread handling and spraying of chemical substances [7]. The products are directly injected into the tree, minimizing the risk of dermal contact or inhalation by the applicator. Moreover, endotherapy reduces the risk of public exposure to pesticide residues [3]. Since the plant protection products are contained within the tree’s vascular system, the potential for residues to drift onto unintended surfaces or remain on the exterior of the plant is substantially lower compared to spraying [17]. Studies on fruit trees treated with trunk injections have shown that while the active ingredients can be found in the fruits, the residue levels at harvest are often below the Maximum Residue Levels (MRLs) defined by authorities [13]. For example, in apple trees, residues have been reported to persist at levels significantly below the MRL of 3 ppm established by the US EPA [23]. In cherry trees, injecting 0.56 g of acetamiprid into the trunk provided more than 95% control of *Rhagoletis cerasi* while keeping residues below permitted levels [24]. Acetamiprid and sulfoxaflor were detected at maximum concentrations of 0.047 mg/kg and 0.006 mg/kg, respectively, in pine nuts. These values remained below the MRLs set by Korea, the EU, and the US, and short- and long-term risk assessments indicated negligible risk to consumer health [25]. These accurate checks on tolerable residue thresholds ensure a greater level of safety for consumers.

## 4. Endotherapy Techniques

**Figure 1:**
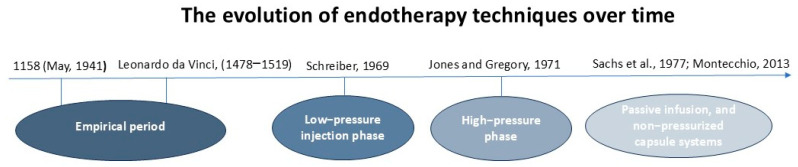
A timeline illustrating the key stages of the evolution of endotherapeutic techniques: from rudimentary manual instruments (drills) to the most innovative methods at present.

First attempts to apply endotherapy on trees were made by exploring methods of trunk injections, delivering chemicals using injecting solutions into a hole made in the tree stem. The injecting mechanism evolved over time through evaluating different solutions of hole creation, methods of injectate delivery, and pressure sources used to supply the injected material, resulting in four phases of development: the empirical period, low-pressure injection stage, high-pressure injection phase, phase of advancements with passive infusion, and non-pressurized capsule systems (Figure 1). Each improvement offered different benefits and limitations in terms of distribution speed, invasiveness, and scalability [6,10].

### 4.1. Empirical Period

The first explorations of the possibility of applying endotheraphic treatments to trees were made in 1158, without a scientific aim [26]. Evidence of the application of endotherapy can be found, as already mentioned, in the writings of Leonardo da Vinci, in which he describes in detail the procedure applied, using a gimlet and a syringe. Then, most arborists referred to this method, plugging the hole with wood to try to ameliorate the injection process and find an application for endotherapy [27]. Among these, Wilson [28], was the first to apply endotherapy as a method to control tree pests and pathogens.

### 4.2. Low-Pressure Injection Phase

Solutions for applying endotherapy to trees increased between the 19th and 20th centuries thanks to advances in botany, plant physiology, and plant anatomy, which contributed to the understanding of how tissues heal after injury (compartmentalization) and how water moves within trees (cohesion–tension theory) [13]. At the basis of the improvement there was, hence, a deeper understanding of the functioning of plants’ vascular system that allowed for the setting up of effective methods that were able to inject solutions and ensure their correct expansion among tree tissues. Indeed, most injection methods developed at this stage were based on gravity-driven injection. Liquid solutions were left attached to the trunk in containers, allowing for prolonged treatment periods via gravity flow [29]. As an example, Shevyrev [30] exposed wood to treatments by placing the surface tissue in contact with the chemicals that were put in waterproof cones around the trunk [26]. Renewed interest in trunk injection surged following outbreaks of major tree diseases, such as Dutch elm disease (*Ophiostoma ulmi*), in the USA during the 1940s, where fungicide injections showed promising results [13]. Schreiber [31] developed an endotherapy method using a plastic bottle connected to a glass medicine-dropper via a rubber hose, while Schwarz and Van Vuuren [32] created equipment that was able to make two injection ports at the opposite sides of the tree. Even if these methods were intuitive and easy to apply, chemical solutions could degrade due to their long storage and continuous flow, which could create a precipitate limiting the effectiveness of treatment [29].

### 4.3. High-Pressure Phase

**Figure 2:**
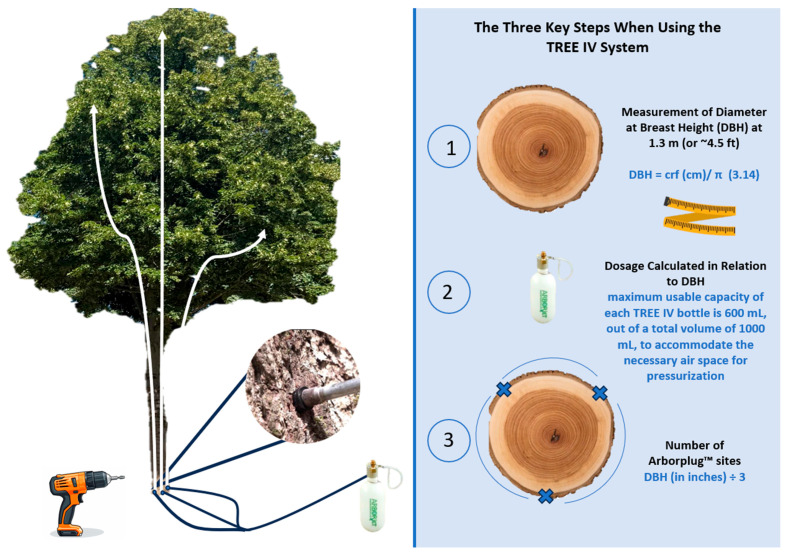
Field photo of treatment on a lime tree (Tilia cordata) in an urban tree-lined avenue with the Tree IV^®^ system, an efficient high-pressure-assisted endotherapy technique that enables precise therapeutic delivery, improved absorption, and optimal control by the operator during application (at 1.3 m DBH). The maximum dose that can be used is 200 mL per injection site, with a maximum of three holes per tree.

This phase is characterized by the application of controlled external pressure to force the therapeutic solution into the tree’s vascular system [20] (Figure 2). The possibility of injecting solutions into trees’ xylematic tissues with pressurized methods started to be explored and high-pressure injection tools were implemented [28,33,34]. Filer [35] and Brown and Bachelor [36] researched the implementation of the technique, drawing attention to its portability, rapidity, user-friendliness, and low-cost features. As a result, lag screw-based injection methods were popularized and assumed great importance in experimental applications [37,38]. Most of these tools were based on gas pressure technology which, combined with a hydraulic cylinder, used compressed gas to inject the treatment solution into the tree trunk. Subsequent changes made pistol-grip devices popular, with most being based on a battery-powered drill and equipped with an injection cylinder [39,40,41,42]. Since the 1990s and 2000s, the increasing spread of invasive pests and new diseases worldwide has further revived research and the application of endotherapy [13]. At present, pressure can be supplied through various means, such as hand pressure, compressed gas, compression springs, elastomers, or pumps [10]. Research indicates that pressurized methods result in faster translocation and higher concentrations of the injected substance in the upper parts of the tree, sometimes requiring smaller volumes compared to non-pressurized techniques [20]. This approach can also lead to reduced labor and more predictable treatment times and is considered more suitable for unstable substances like certain antibiotics. However, the relationship between pressure and phytotoxicity can be complex and substance-dependent [2]. Examples of modern devices used with high-pressure systems include Quick-jet^®^, Tree IV^®^, and Viper^®^. The Quick-jet^®^ system operates at pressures ≥35 psi with a maximum reservoir volume of 600 mL, employing approximately DBH/3 injection sites and achieving uptake within minutes; the Tree IV^®^ system functions at 35–60 psi with a practical maximum of 600 mL, requiring DBH/2 injection sites and uptake typically within 10–45 min; the Viper^®^ assembly serves as the needle–valve interface under the same pressure ranges, delivering per-site doses calculated as total treatment volume divided by the number of injection sites [12,21].

### 4.4. Passive Infusion and Non-Pressurized Capsule Systems

**Figure 3:**
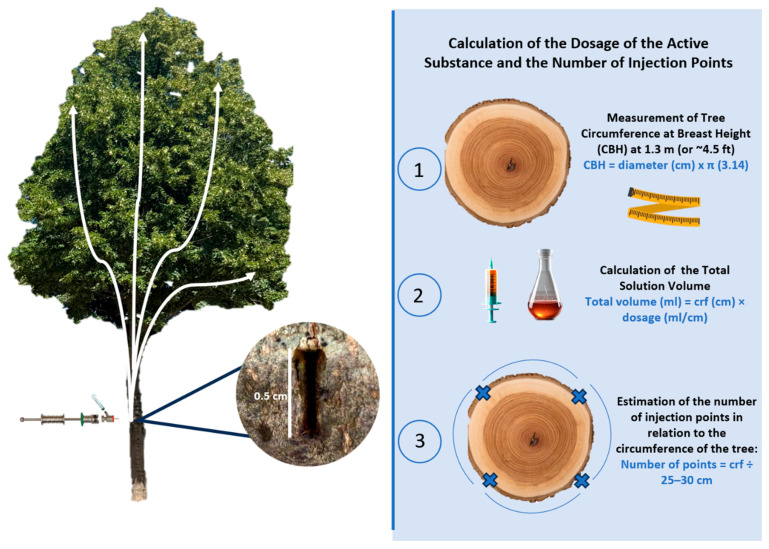
Field photo of treatment on a lime tree (*Tilia cordata*) in an urban tree-lined avenue with the innovative, non-pressurized BITE^®^ system, designed to reduce tissue trauma and the extent of the injury caused by blade insertion (at 1.3 m CBH), while promoting passive sap uptake. The dose depends on the formulation used and the number of holes is calculated using the formula nh = circumference/25~30 cm).

**Figure 4:**
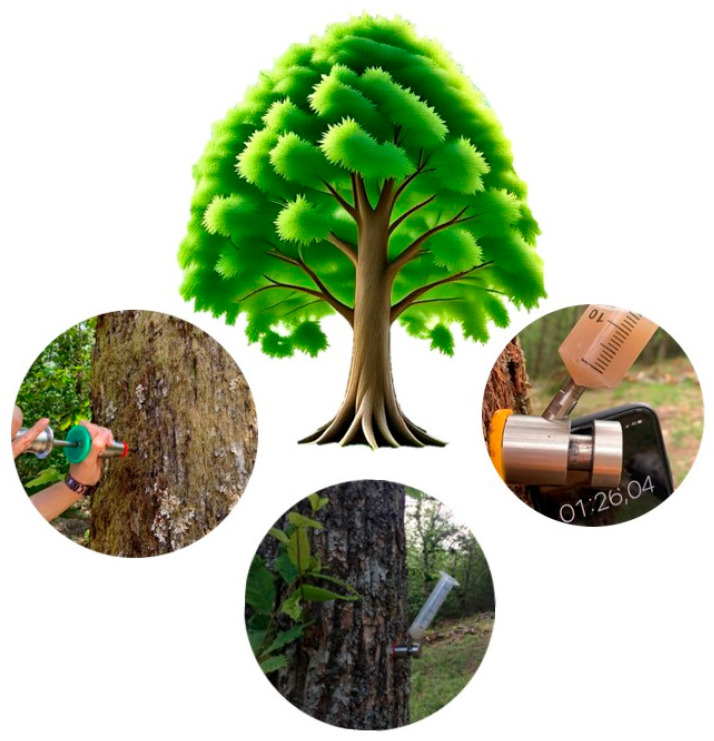
Field photo of endotherapy treatments in a chestnut (*Castanea sativa*) orchard with the BITE^®^ system for the control of the chestnut brown rot agent *Gnomoniopsis castaneae* using *Trichoderma*-based biocontrol agent (BCA) formulations.

A step forward was made with the possibility of coupling passive infusion with endotherapy systems. This foundational approach relates to the concept of passive infusion, defined as the uptake of applied chemicals primarily dependent on atmospheric pressure and the natural sap flow (driven by evapotranspiration) within the tree’s xylem [43,44]. Non-pressurized techniques are a category within this evolution, involving the introduction of solutions without the application of significant external force, leveraging the tree’s inherent capacity for absorption [1,6]. While conceptually simple and potentially causing fewer phytotoxic responses or harmful vessel cavitations [2], these methods can be slow and may require larger entry points or leave open wounds, increasing the risk of the tree becoming infected with wound parasites [21]. Significant progress was made by the improvement of delivery methods, allowing damage to the trunk to be reduced and requiring little drilled wounds (3–5 mm) to be effective [13,17,22]. Among these, devices based on capsule systems can function under low or hand pressure, such as certain Mauget^®^ systems [13], or can be self-dispensing to provide a constant, low-pressure delivery into the injection port [17]. These devices fall under the category of microinfusion/microinjection systems, designed to deliver small volumes (5–120 mL per capsule injection) of product directly into the vascular system [6]. Systems like Fertinyect^®^ capsules provide a contained method for introducing a specific volume of product into a drilled hole under controlled pressure (0.5 bar–approx. 50 kPa) [6]. The continued development includes innovative non-pressurized, drill-free systems like BITE^®^ (De Rebus Plantarum, Vicenza, Italy) (Figure 3 and Figure 4), which are designed to minimize injury while still relying on a passive uptake potentially enhanced by mechanical effects [10,44]. Indeed, this method relies on special blades able to open lenticular shaped slots that, coupled with the possibility of exploiting a possible Venturi effect for natural uptake, are able to administer the active ingredient via natural uptake [10,44].

## 5. Endotherapy Application

Basically, endotherapy functions by applying products directly into the tree’s vascular system, specifically the xylem [1]. The injected substance is absorbed and distributed throughout the tree primarily by exploiting its normal absorption capacity, moving with the xylem sap flow that rises from the roots to the leaves, driven by the plant’s transpiration pull [20]. The physical act of creating an injection point in the trunk is supposed to induce a Venturi effect, where the temporary reduction in vessel cross-section causes sap speed to increase and pressure to decrease, facilitating passive absorption, especially when sap flow is high [44]. Low external pressure can also be applied to force uptake. For the treatment to be effective, the applied products, especially systemic ones, must be capable of translocating within the plant to reach the target location [6]. The efficiency and extent of this translocation and distribution are significantly influenced by the physicochemical properties of the active ingredients and the formulation itself, including factors like molecular mass, water solubility, lipophilicity (Kow), polarity (pKa), pH, viscosity, and stability [2]. Commercial formulations designed for conventional methods are often not compatible with vascular transfer, highlighting the need for specific formulations for endotherapy [45]. The process is also profoundly influenced by the plant’s own morphology and physiology, as xylem anatomy, seasonality, and formulation compatibility with vascular tissues significantly influence the plant’s ability to absorb and distribute active ingredients [6]. Key factors include the following: (1) the species characteristics, which necessitate adapting techniques and potentially equipment for different types of trees (e.g., palms vs. deciduous trees); (2) the trunk structure and vitality, as healthy trunks with good structure are better suited to these applications; (3) the tree’s age, as demonstrated by challenges in treating old or centenarian trees, possibly due to natural vessel occlusion hindering uptake; (4) and the presence of xylem vessel occlusion, which can be due to aging, cavitation, or disease [22]. Furthermore, the application technique itself, including factors like the presence or absence of drilling, the location, depth, angle, and diameter of the injection hole, and the needle shape, impacts uptake [13]. Damage caused by traditional drilling methods can negatively affect the functionality of adjacent woody tissues and delay wound closure, potentially impacting sap flow and product distribution [46]. Advanced injection systems aiming to minimize damage to woody tissues, particularly those employing drill-free techniques or lenticular-shaped blades, are often preferred to minimize damage to woody tissues and promote faster wound healing compared to traditional drilling methods [13]. Pressurized methods can offer faster translocation and predictability, while low/no-pressure methods may pose less risk of phytotoxicity or vessel cavitation [10]. The timing and method of administration are interdependent and depend on the specific active ingredient and the tree’s physiological stage, particularly sap flow and transpiration rates, to ensure the compound reaches the target area at the optimal concentration during the period of highest pest or pathogen pressure [46]. Ideally, injections should occur during periods of active sap flow and coincide with peak pest or pathogen vulnerability [1]. Hence, application during active transpiration is crucial to facilitate product movement within the xylem, correlating with the circulation of water and nutrients during the photosynthetic cycle. Additionally, adverse environmental conditions, such as drought or pre-existing vascular damage, may impair absorption and increase the risk of embolism or phytotoxicity. Hence, understanding these biological and physical interactions is fundamental for optimizing an endotherapy procedure.

### 5.1. Products Formulation

**Figure 5:**
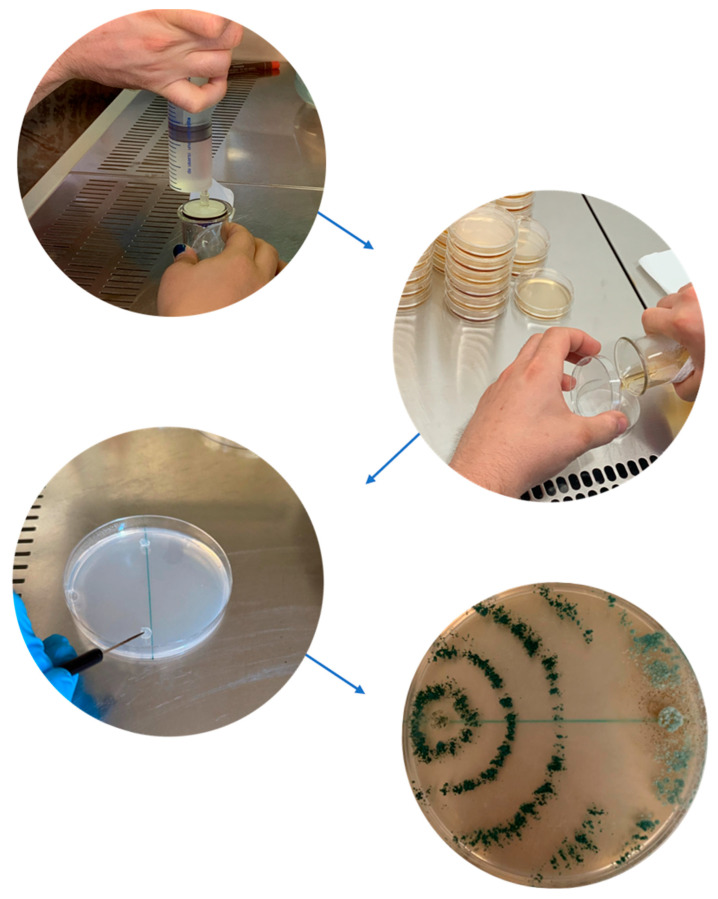
Flowchart of the main laboratory steps in a dual-culture antagonism assay of a *Trichoderma* spp. biocontrol agent (BCA) against a plant pathogenic fungus. The image at the bottom clearly shows the greenish concentric halos formed by *Trichoderma* sp., which colonized the space and nutrients in the Petri dish before overgrowing the target pathogen upon contact [19].

The selection of the active ingredient class and its specific formulation is paramount for effective endotherapy application, requiring systemic properties and suitability for injection, as classical agrochemical formulations may be inappropriate or even detrimental [2,47]. A wide range of active ingredient classes can be delivered via endotherapy, including insecticides, fungicides, antibiotics, nutrients, fertilizers, growth regulators, biostimulants, biopesticides, and defense activators [13]. Among the most commonly used insecticides are emamectin benzoate, imidacloprid, thiamethoxam, and abamectin, which have proven effective against pests such as the red palm weevil (*Rhynchophorus ferrugineus*) and the pine tortoise scale (*Toumeyella parvicornis*) [6,12,15]. Fungicides and related compounds like phosphonate, cyproconazole, and Dentamet^®^ (zinc and copper complexed with citric acid) are applied via endotherapy, with devices like BITE^®^, Arbocap^®^ (Ital-Agro, Turin, Italy), Chemjet^®^ Tree Injectors (Logical Result LLC, Interlochen, MI, USA), and various types of drill. These are used to manage several fungal, pseudofungal, and bacterial diseases, including those caused by *Phytophthora* species, certain grapevine wood pathogens (like *Phaeomoniella chlamydospora*), and *Xylella fastidiosa* [4,21,22,48,49,50,51]. Trunk injections of antibiotics, notably terramycin and penicillin G, using Chemjet^®^ are used to combat bacterial diseases like fire blight (*Erwinia amylovora*) and lethal yellowing diseases in palms [5]. By offering a direct route for delivering these kinds of formulation into the tree’s vascular system, endotherapy fundamentally impacts both the distribution and persistence of these active ingredients compared to conventional applications. Bypassing external barriers like the cuticle and soil, endotherapy treatments can potentially lead to higher concentrations reaching internal targets [52]. Indeed, studies have shown that injected substances can be detected in canopy tissues like leaves and twigs, confirming their distribution [12,15]. Concerning persistence, some formulations specifically designed for endotherapy, such as the emamectin benzoate and Revive^®^ products, are noted for their high persistence in several tree species (conifers, willows, ash, and palm), potentially lasting up to a year, significantly reducing the frequency of treatments required [10,13,14,15,53]. Oxytetracycline also appears to have increased stability and long-term efficacy when injected compared to foliar sprays, and its uptake and persistence has been demonstrated in *Citrus* spp., palms, and almond tissues [5]. However, while some products show long persistence, others, like endotherapy abamectin, may have a more limited duration, not always guaranteeing complete distribution of the injected substance throughout the entire plant and therefore potentially limiting the long-term efficacy of the treatment [53]. Persistence is largely governed by the conditions of the endotherapic method, which provides a physical barrier against environmental breakdown. For highly effective compounds like emamectin benzoate, this protection translates into measurable residue concentrations capable of providing disease or pest control for up to a year or longer, depending on the tree species and targeted pest [13,14,15]. For products like oxytetracycline, injection counters its inherent molecular instability, leading to long-term efficacy against vascular pathogens. In addition, establishing the optimal time of year for injection (e.g., spring versus fall) is important for predicting efficacy and compound residue, particularly in commercial production [5]. For these reasons, the need to research new formulations with improved bioavailability (translocation and persistence within the plant) is becoming important [12]. In addition, the extensive or improper use of chemically active ingredients, particularly antibiotics and pesticides, raises concerns about the development of resistance in target organisms [54] (see Section 6). The use of eco-friendly formulations is viewed as a promising strategy to address these issues. New product strategies are focusing on developing bioformulations to replace antibiotics and pesticides for endotherapy application [15,16,17,22]. The research has explored agents such as nanoparticle-based products, which could potentially be more potent and offer advantages like improved stability, systemic action, and slow release for longer-lasting effects [55,56,57]. Beyond formulation chemistry, the nature of the active ingredient itself is evolving towards more sustainable options. This includes the use of biocontrol agents (BCAs), such as fungi and bacteria, or natural products like plant extracts, essential oils, or antimicrobial peptides, applied via endotherapy [16,19,58,59,60]. Studies have evaluated the use of biocontrol strains like *Trichoderma* spp. for controlling pathogens in chestnut trees via endotherapy [19]. A crucial step before using BCAs in the field is their proper assessment for biocontrol potential. This can be performed in the laboratory using dual culture bioassays (Figure 5).

Other biocontrol trials assessed peptides for managing *Xylella fastidiosa* in model plants and almond [56,61], ozonated water in woody tissues of grapevines to control fungi (e.g., *Phaeoacremonium aleophilum*) associated with esca disease [1], phenolic extracts from olive leaves against *Xylella fastidiosa,* and essential oil components [22]. The application of these kinds of formulations through endotherapy is seen as a way to reduce the risk of the development of resistance in target organisms, attributed to the diverse mechanisms of action inherent in biological or natural agents (e.g., their ability to stimulate the plant’s own defenses) compared to chemical products, or to the reduced dispersal and reduced contact with non-target organisms compared to broad applications [62]. Furthermore, the delivery of beneficial substances such as growth regulators, defense activators, plant biostimulants, fertilizers, and nutrient solutions like seaweed extract and iron amino chelate contributes to improved tree health and addresses nutritional deficiencies [2,63,64,65]. However, achieving a homogeneous distribution of injected compounds within the tree using bioformulations remains a concern and poor distribution, mainly tied to the physical or chemical properties of the biocontrol agent, could favor the adaptation and tolerance (a form of resistance) of the target organisms. One clear example involves large molecules: the injection of the Blad-containing oligomer (BCO), a high-molecular-weight oligomer (210 kD) tested against grapevine pathogens, was deemed unsuccessful because the size of the compound likely prevented the solution from entering the xylem vessels of the plants [4]. Similarly, while the antimicrobial peptide BP178 demonstrated strong bactericidal activity in vitro against bacteria such as *Xylella fastidiosa*, its efficacy in planta was hindered by the uncertainty of its distribution within the plant’s vascular tissue [16]. Such non-uniform or insufficient distribution results in concentrations in certain parts of the plant canopy becoming too weak, creating tolerant hotspots where pathogens are exposed to sub-lethal doses, enabling them to adapt and potentially develop resistance or tolerance [13,66]. Therefore, optimizing the formulation of these eco-friendly agents is essential to ensure effective distribution, thereby enhancing their bioavailability and mitigating the risks of resistance and adaptation (Figure 5). In addition, the Reduction In Resistance risks in bioformulation-based treatments often fails to acknowledge the critical role of tolerance in the efficacy of these new sustainable compounds. While the traditional extensive application of pesticides and antibiotics, especially without criteria, poses a major problem in creating pathogen resistance [6], many bioformulations function by eliciting plant defense responses, leading to induced systemic resistance (ISR) or systemic acquired resistance (SAR) [16,57]. This mechanism grants the host a higher level of tolerance toward pathogens [62]. For example, studies using bifunctional peptides (such as BP178) or essential oils (such as EGL2) against pathogens such as *Xylella fastidiosa* have shown that a reduction in disease severity is associated with both direct antimicrobial action and activation of the host immune system [16,62]. Notably, the transient expression of peptides in some plants resulted in reduced disease severity, even though the pathogen population levels did not decrease compared to non-treated controls, suggesting the plant acquired tolerance rather than achieving complete eradication [57]. Therefore, when evaluating the success of biological control agents (BCAs), it is essential to consider the dual mechanism of action and the resulting tolerance, alongside the reduced risk of traditional resistance development [62].

### 5.2. Digital Technologies for Endotherapy

Future developments in vegetative endotherapy extensively envision the integration of automated and robotic application systems to enhance efficiency and precision, particularly for large-scale commercial applications where current manual methods pose significant challenges due to their slow speed and substantial labor requirements [67]. The digital transformation and automation revolution are expected to accelerate the creation of such systems. Examples of these envisioned technologies include new technologies for robotic applications and artificial intelligence (AI), using sensors to detect, prevent and treat diseases and pests, alongside high-efficiency automatic endotherapy equipment designed to replace long hours of physically demanding and repetitive work [6]. Robotics are also seen as impacting precision horticulture beyond automated fruit collection, potentially leading to holistic trunk management platforms that could be applied in cultivations like olives [2,68]. An automated trunk injection system prototype that is drill-free and pressurized has been developed for rapid injection [67]. This system features a retractable positioning arm, an end effector with a simultaneous multi-puncture mechanism, and a metering pump with adjustable pressure and flow rate [66]. Furthermore, an AI-enhanced sensing system is under development to detect the tree trunk and desired injection point in real-time, and to estimate tree canopy volume to inform the precise dosage [69,70]. Sensors like flow meters and solenoid valves that can deliver a fixed volume to each injection port would also aid in achieving automation and homogeneous distribution [64]. Ultimately, the development of autonomous injection systems capable of communicating with each other is considered a promising strategy for achieving accelerated large-scale trunk injection [71]. This could involve a fleet of AI-enabled automated or fully autonomous systems to reduce injection time and application costs in large orchards, requiring high-level multi-robot coordination [10]. These advancements are integral to the concept of “precision agriculture” and the “orchard of the future,” aiming for a more efficient, smart and sustainable approach to tree cultivation and integrated pest management [2]. However, most of them currently consist of a mix of active prototypes under development or advanced proprietary systems that represent the future direction of endotherapy, rather than highly efficient and widely marketed automatic endotherapy equipment. The main goal is to make endotherapy deployable at a speed comparable to conventional methods, ensuring precise and efficient dosage delivery and minimizing tree wounding, especially through non-drilling techniques facilitated by automation [24]. However, digital technologies could also be used to understand the spatial distribution and temporal persistence of active ingredients within plant tissues, which directly influence their bioactivity and the efficacy of treatments [72]. While analytical techniques applied on petioles, leaves, phloem, and xylem sap such as LC-MS/MS have been used to study compound concentrations in tissues over time, collecting samples from 6 to 128 h after injection treatments [5,7,13,73], and methods like Confocal Laser Scanning Microscopy with fluorescence quantification have visualized movement in plant sections [51], digital technologies are becoming increasingly pivotal in monitoring and optimizing endotherapy treatments, facilitating more precise and effective plant health management. In this connection, Ferreira et al. [21] formulated a mathematical model to simulate the translocation dynamics of cyproconazole in palm trees, evaluating diverse application methodologies and establishing predictive frameworks to refine dosage protocols. Complementing the work of Ferreira et al. [21], Chihaoui-Meridja et al. [15] conducted temporal analyses of insecticide residues in palm leaves following injection, yielding critical data on residue degradation patterns and treatment longevity. Further advancing this field, Moll et al. [16] investigated the retention profiles of an antimicrobial peptide in almond tree vascular system post-endotherapy, providing mechanistic insights into the absorption and systemic translocation of bioactive compounds. The convergence of such empirical findings with advanced digital tools—including sensor networks and artificial intelligence (AI)-driven analytics—has transformative potential. These technologies enable the real-time, high-resolution tracking of active ingredient distribution, thereby supporting data-driven interventions that optimize therapeutic outcomes while reducing agrochemical waste and environmental footprint.

### 5.3. Efficacy of Endotherapy

Extensive research supports endotherapy as a precise and sustainable phytosanitary strategy for managing tree diseases and pests (Table 1). This technique has been applied across diverse tree species, including apple (*Malus domestica*), citrus (*Citrus* spp.), chestnut (*Castanea sativa*), mulberry (*Morus* spp.), almond (*Prunus dulcis*), olive (*Olea europaea*), palm (*Arecaceae*), and pine (*Pinus* spp.), to mitigate a wide range of biotic stressors. Its application in plant pathology is supported by several studies demonstrating its efficacy against various pathogens [21]. Collectively, these studies highlight the adaptability of endotherapy across a broad taxonomic and ecological spectrum, underscoring its potential as a transformative alternative to conventional phytosanitary treatments, aligning pest control efficacy with the principles of environmental sustainability. For instance, Gyuris et al. [24] confirmed the efficacy of trunk-injected insecticides against *Rhagoletis cerasi* in cherry production, while Archer et al. [5] demonstrated the prolonged antimicrobial activity of oxytetracycline in citrus cultivars affected by Huanglongbing (HLB). Endotherapy treatments with bifunctional peptides have been shown to reduce pathogen population levels and disease severity of the bacterial pathogen *Xylella fastidiosa* in model plants like *Nicotiana benthamiana* [57] and almond plants [16,61]. Treatment with Dentamet^®^ also led to reduced *X. fastidiosa* severity and cell densities in olive trees, simultaneously improving their vegetative status [51]. Trunk injection of phenolic extracts from olive leaves and solutions based on garlic powder and potassium phosphite produced bacteriostatic effects against *X. fastidiosa* in vitro and stimulated vegetative growth of infected olive trees [22]. Endotherapy has also been explored for the biological control of fungal and pseudofungal diseases. Berger et al. [74] tested the microbial agents *Trichoderma* spp. and *Bacillus amyloliquefaciens* against *Phytophthora* spp. Phosphite trunk injection is a common practice against *Phytophthora* species [50] and has demonstrated effectiveness against Chestnut ink disease [49] and avocado root rot [75]. Trunk-injected systemic fungicides were effective in managing Dutch elm disease (*Ophiostoma ulmi*) [13] and inhibited fungi like *Armillaria tabescens* with propiconazole [76]. Endotherapy treatments proved effective in suppressing *Gnomoniopsis castaneae* in chestnut trees, showing reduced incidence of brown rot on fruits [19] (Table 1). Various substances, including botanical pesticides like carvone, citral, and thymol, along with potassium phosphite and Tecto 20S, were tested for efficacy against the fungus *Geosmithia morbida* in walnut trees. Furthermore, trunk injections of antibiotics such as oxytetracycline [77,78] and penicillin G [54] and plant defense activators were applied for the control of citrus huanglongbing, caused by the bacterium *Candidatus* Liberibacter asiaticus [71,79,80]. Dal Maso et al. [81] tested endotherapy treatments with allicin and Thiabendazole against Ash Dieback Disease, caused by the pathogen *Hymenoscyphus fraxineus*. While neither allicin nor Thiabendazole completely stopped the growth of the pathogen, both treatments significantly slowed down the development of cankers. The approach has further proven effective in insect pest management, with Kavallieratos et al. [14] achieving near-complete suppression of *Xylotrechus chinensis* in mulberry trees and Pal et al. [11] documenting significant population declines in *Paysandisia archon* in palms following imidacloprid injections. Additionally, Di Sora et al. [12] established endotherapy as an effective strategy against *Toumeyella parvicornis* infestations in Mediterranean pines.

## 6. Drawbacks of Endotherapy

**Figure 6:**
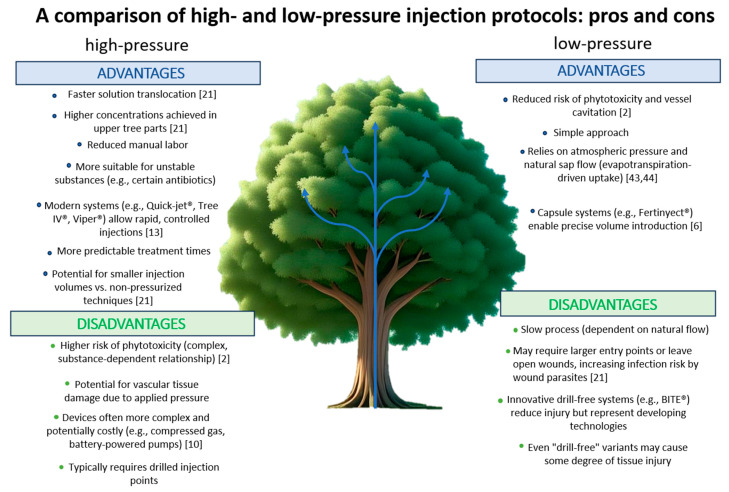
Overview comparing two endotherapy methods, emphasizing the key differences in technique, efficiency and safety and providing a detailed examination of their respective advantages and limitations.

Despite the multiple advantages of endotherapy for plant protection from a sustainability and environmental protection perspective, several drawbacks limit its widespread adoption and effectiveness for managing plant diseases and pests. A significant concern is the damage caused to tree tissues, particularly with traditional drilling methods, which can lead to loss of woody tissue functionality, delayed wound closure, and infection by microorganisms that attack wood, resulting in internal decay and compromised structural strength and stability [6,43]. Injecting solutions into trees thus requires methods that minimize damage to woody tissues during injection [83]. Studies have also noted side effects like larger necrotic areas in treated branches compared to controls, although the overall curative efficiency results were not always statistically significant [13]. Furthermore, these methods risk inducing phytotoxicity and harmful vessel cavitations, potentially causing embolism or necrosis in susceptible plants [2]. In addition, in urban and suburban environments, which often feature tall trees and high-density human populations, the application of plant protection products faces specific challenges and regulatory constraints [13]. For instance, supranational rules like the European Pesticide Regulation (EC) No. 1107/2009, and specific national laws, such as the National Action Plan (PAN) in Italy, explicitly prohibit the application of active ingredients in urban settings [12]. Regulatory frameworks, such as the EU Directive (EC) 2009/128, promote the sustainable use of pesticides, aiming to reduce risks and impacts on human health and the environment while encouraging integrated pest management and non-chemical alternatives [19]. Outside Europe, the regulatory landscape demonstrates regional acceptance of the necessity for endotherapy, particularly in combating systemic threats. As an example, in North America, the use of Plant Protection Products (PPPs) via techniques such as trunk injection is generally well-accepted and implemented within the commercial fruit production sector and the larger tree care industry [84]. In this context, endotherapy is often viewed as a promising alternative because it delivers products directly into the tree’s vascular system, significantly reducing environmental contamination, eliminating spray drift, and minimizing exposure risks to users and the public compared to conventional methods [47]. However, for safe and effective endotherapy application, several guidelines and considerations are important: (1) it is crucial to select the appropriate technique based on factors like tree anatomy, health condition, and environmental conditions, on which the effectiveness of treatments is highly dependent [6]; (2) it is necessary to minimize damage to tree tissues during the injection process, particularly avoiding issues like loss of woody tissue function, delayed wound closure, and decay associated with traditional drilling methods [46]; (3) proper application, including the number and positioning of injection points, is vital for adequate distribution and post-application; (4) the sealing of injection points is recommended to prevent the proliferation of microorganisms and pests [13]; (5) when using pressurized systems, depressurization after installing the plug is essential to avoid airlocks that could lead to cracks in the bark or embolism within the tree [10]. Hence, hiring well-trained professionals is crucial for safely and efficiently performing endotherapy applications [15]. Furthermore, endotherapy requires the individual treatment of each tree, which can be time-consuming and may not be practical or cost-effective for large-scale applications like nurseries or young vineyards [4]. As an example, non-pressurized methods, relying on natural uptake, can sometimes take 60–70 min for absorption in healthy plants, but cases where over 15 h was required have been reported for sick plants or particularly unsuitable conditions [6,20]. Regarding cost-effectiveness, the expenses associated with high tree density can lead to an increase in costs, requiring a long period of time and high labor input [13]. Indeed, in environments like large-scale nursery production systems or young vineyards, the process of injecting chemicals is explicitly noted as time-consuming and potentially impractical [4]. The technique may also be unsuitable for certain trees, such as old or centenarian trees, particularly those with a particularly twisted trunk (e.g., some olive trees), possibly due to natural vessel occlusion that hinders product uptake and distribution [26]. Hence, there is still a need to optimize application techniques and address these biological and practical challenges for endotherapy to achieve its full potential. There is also a recognized need for the development of specific product formulations designed for endotherapy, which are still lacking, constituting a limit to the application of this method [6]. Another significant shortcoming of endotherapy treatments is the risk of generating resistance in pathogens or pests if active ingredients are applied extensively without proper criteria [80]. For instance, in controlling the red palm weevil, the rotation of active ingredients with different modes of action is recommended to avoid the development of insecticide resistance [15]. Additionally, Scortichini et al. [51] demonstrated that compounds like Dentamet^®^ can influence vegetative health, as measured via NDVI, underscoring the need to balance phytosanitary benefits with physiological impacts. There are also concerns about the synthesis of certain compounds potentially inducing tylose overproduction or other gene products that could exacerbate disease symptoms [57]. Furthermore, there are concerns regarding potential interactions with the plant’s endophytic community, whose beneficial component is important for tree health [84]. Indeed, interactions between injected substances and native endophytes could alter microbial composition and dynamics, affecting the health of trees and, consequently, the ecosystem services they provide. While endotherapy is often preferred for reducing the environmental dispersion of active ingredients compared to spraying [12], the substances injected, such as abamectin, still have known ecotoxicological effects, which could potentially impact non-target animal species and broader biodiversity, particularly microbial communities within the tree [53]. The compatibility of endotherapy with sustainable management frameworks depends on mitigating collateral harm to non-target species, preserving biodiversity, and preventing ecological disruption through evidence-based precision application methods. Addressing these multifaceted risks is essential to align endotherapy with holistic, environmentally responsible tree care practices (Figure 6).

## 7. Future Perspectives

Endotherapy emerges as a promising innovation in arboriculture and plant protection, offering a potentially vital and evolving approach to tree care and defense [6,85]. Its benefits include reduced pesticide drift, preservation of beneficial organisms, and enhanced precision in treating pests and pathogens inside the tree, vascular parasites in particular. The method promises significant advancements, especially for counteracting challenges like invasive pests, emerging diseases, and the imperative need for more sustainable control practices [13,21]. However, the efficacy and applicability of endotherapy are intrinsically linked to the substances being injected and the techniques employed for their delivery into the tree’s vascular system [81,86,87]. Future research and development priorities should focus on the following:

- Ameliorating the distribution uniformity;
- Applying less invasive, low-injury delivery methods;
- Refining injection technologies through more efficient operational throughput/automated dosing;
- Expanding the range and type of active ingredients used, moving towards more environmentally friendly options.

Promising avenues for novel active ingredients include (1) bioformulations and Biocontrol Agents (BCAs), employing living microorganisms or their products; (2) novel peptides and proteins; (3) nanotechnologies-based products that are considered strong candidates for future endotherapy applications; (4) phytochemicals and botanical extracts, i.e., natural compounds derived from plants being explored as biopesticides or plant health enhancers; (5) ozonated water [2]. Parallel to the search for new substances, advancements in delivery methods are critical, requiring further research to fully understand the distribution and persistence of injected products within different tree species and under varying environmental conditions [10,22]. However, critical limitations persist, including risks of pathogen resistance and potential disruption to endophytic communities essential for tree resilience [84]. The integration of endotherapy into sustainable arboriculture and urban tree care practices hinges on its compatibility with agro-ecosystem integrity and biodiversity conservation. Indeed, endotherapy is increasingly assessed not as a standalone treatment but as a critical, potentially synergistic component within broader, integrated tree management strategies, recognized as a valuable tool in helping trees adapt to and survive the impacts of climate change, which can stress them and favor the proliferation and geographic expansion of pests and diseases [2]. While it addresses urgent phytosanitary challenges, its long-term success requires balancing efficacy with environmental stewardship. Therefore, by combining endotherapy with other tree care methods, whether agronomic (e.g., soil harrowing, pruning, fertilization, mulching and sanitation), biological (e.g., biocontrol agents, plant-based active ingredients and defense elicitors) or chemical (e.g., foliar fungicide and insecticide treatments), it is possible to improve the overall effectiveness of tree health management, increase sustainability, and address the complex factors that influence tree health [15,17,51]. Integrated approaches acknowledge the necessity of managing tree health holistically, considering tree anatomy, physiology, health status, and environmental conditions, when selecting injection methods and substances. Among the future directions in integrated management, the following can be listed: (1) Resistance Management Strategies such as rotating the different active chemicals or modes of action applied by endotherapy [15]; and (2) Precision Agriculture and Data Integration, embedding endotherapy within a “smart data umbrella” using sensors and AI [2,6]. Additionally, multidisciplinary studies on biodegradable formulations, resistance management strategies, and precision delivery systems could enhance its safety and scalability. Realizing these promising future perspectives will necessitate ongoing investment in research, standardization of methodologies, and robust collaboration among scientists, industry, and tree care professionals. Collaboration between researchers, arborists, and policymakers will be essential in refining endotherapy as an integrated, ecologically responsible approach to tree care, ensuring its role in fostering resilient woody crops and urban forests for future generations.

## Figures and Tables

**Table 1:** Lists of endotherapy protocols used to control various plant parasites (fungi, oomycetes, bacteria, and insects), with percentages of treatment effectiveness.

Agent Name	Product Formulation	Host	Endotherapic Treatment Efficacy	Reference
Fungi and oomycetes				
*Armillaria tabescens*	Propiconazole	*Prunus persica*	Evaluated only in vitro	Amiri et al. [77]
*Geosmithia morbida*	Tiabendazole, Procloraz, Allicin, Tetraconazole	*Juglans nigra*	28–31%	Dal Maso et al. [65]
*Gnomoniopsis castaneae*	*Trichoderma* spp.	*Castanea sativa*	26–40%	Benigno et al. [19]
*Hymenoscyphus fraxineus*	Allicin, Thiabendazole	*Fraxinus* spp.	56–67%	Dal Maso et al. [81]
*Ophiostoma ulmi*	2-(3′,4′-dichlorophenol)-1,3-dioxolan-2-ylmethyl)imidazole	*Ulmus* spp.	44%	Wilson et al. [41]
*Phaeoacremonium aleophilum*	Ozonated water	*Vitis vinifera*	50%	Pierron et al. [82]
*Phaeoacremonium minimum*	Blad-containing oligomer (BCO), Elemental silver, Fosetyl-Al, Glutaraldehyde, Hydrogen peroxide	*Vitis vinifera*	0–90%	Del Frari et al. [4]
*Phaeomoniella chlamydospora*	Blad-containing oligomer (BCO), Elemental silver, Fosetyl-Al, Glutaraldehyde, Hydrogen peroxide	*Vitis vinifera*	0–90%	Del Frari et al. [4]
*Phytophthora* spp.	Phosphite, *Trichoderma* spp.; *Bacillus amyloliquefaciens*	*Quercus robur*; *Fagus sylvatica*	31–86%	Berger et al. [74]
*Phytophthora cambivora*	Potassium phosphite	*Castanea sativa*	Up to 90%	Gentile et al. [49]
*Phytophthora cinnamomi*	Potassium phosphite, metalaxyl	*Castanea sativa, Macadamia* spp.	Up to 90%	Gentile et al. [49]; Akinsanmi and Drenth [50]
Bacteria				
*Candidatus* Liberibacter asiaticus	Oxytetracycline	*Citrus* spp.	c.a 15% reduction in fruits drop	Archer et al. [5]
*Erwinia amylovora*	*Eucalyptus* essential oil	*Pyrus* spp.	30–39%	Montesinos et al. [62]
*Xylella fastidiosa*	Synthetic peptides	*Nicotiana benthamiana, Prunus dulcis*	70–74%	Barò et al. [57]; Moll et al. [16]; Moll et al. [61]
	Zinc and copper complexed with citric-acid hydracids (Dentamet^®^)	*Olea europaea*	Decrease in pathogen DNA concentration (from 27 ng in untreated samples to 3 ng in treated samples)	Scortichini et al. [51]
	*Eucalyptus* essential oil	*Prunus dulcis*	52–68%	Montesinos et al. [62]
	Phenolic extract from olive leaves	*Olea europaea*	Increase in leaf area index (2–10%) and leaf area density (6–9%)	Vizzarri et al. [22]
Insects				
*Cameraria ohridella*	Imidacloprid, Abamectin, Avermectin	*Aesculus hippocastanum*	Up to 82%	Ferracini and Alma [20]; Pal et al. 2018 [11]
*Rhagoletis cerasi*	Acetamiprid	*Prunus* spp.	95%	Gyuris et al. [24]
*Rhynchophorus ferrugineus*	Emamectin benzoate, Thiamethoxam, Imidacloprid, Clothianidin, Fipronil	*Phoenix canariensis*	20–80%	Chihaoui-Meridja et al. [15]; Di Ilio et al. [7]; Gomez and Ferry [47]
*Toumeyella* *parvicornis*	Abamectin	*Pinus* spp.	From 67 (untreated) to 20 (treated) individuals recorded at the peak of population	Di Sora et al. [12]
*Xylotrechus chinensis*	Spirotetramat, Fipronil, Imidacloprid, Abamectin	*Morus* spp.	8–85%	Kavallieratos et al. [14]

The metrics are reported as in the sources and are not comparable between rows.

## Data Availability

No new data were created in this study.
